# Effectiveness of an Educational Intervention in Reducing New International Postgraduates’ Acculturative Stress in Malaysian Public Universities: Protocol for a Cluster Randomized Controlled Trial

**DOI:** 10.2196/12950

**Published:** 2020-02-27

**Authors:** Musheer Abdulwahid Al-Jaberi, Muhamad Hanafiah Juni, Hayati Kadir Shahar, Siti Irma Fadhilah Ismail, Murad Abdu Saeed, Lim Poh Ying

**Affiliations:** 1 Department of Community Health Faculty of Medicine and Health Sciences Universiti Putra Malaysia Selangor Malaysia; 2 Department of Psychiatry Faculty of Medicine and Health Sciences Universiti Putra Malaysia Selangor Malaysia; 3 English Department Onaizah College of Sciences and Arts Qassim University Qassim Saudi Arabia

**Keywords:** acculturative stress, acculturation, international students, adjustment, protocol, cluster, randomized controlled trial

## Abstract

**Background:**

Universities around the world, including Malaysia, have attracted many international students from different countries. Research has reported that acculturative stress resulting from international students’ attempts to adjust to the cultures of host countries is one of the most challenging issues that affects their lives in general and academic lives in particular.

**Objective:**

This study aims to examine the effectiveness of an educational intervention on acculturative stress among new postgraduate international students joining Malaysian public universities.

**Methods:**

A cluster randomized controlled trial design with Malaysian public universities as the unit of randomization will be used in this study. Public universities will be randomized in a 1:1 ratio to be either in the intervention (educational program) or control group (waiting list). Participants in the intervention group will receive 7 sessions in 9 hours delivered by an expert in psychology and the researcher. The control group will receive the intervention once the 3-month follow-up evaluation is completed.

**Results:**

The data will be analyzed using the generalized estimation equation with a confidence interval value of 95%; significant differences between and within groups are determined as *P*<.05. The results of the study underlie the effectiveness of educational program in decreasing acculturative stress of new international students and enabling them to cope with a new environment. The results of this study will contribute to previous knowledge of acculturative stress, acculturation, and adjustment of international students. Furthermore, such results are expected to play a role in raising university policy makers’ awareness of their postgraduate international students’ acculturative stress issues and how they can help them avoid such stress and perform well in their academic life.

**Conclusions:**

We expect that the intervention group will score significantly lower than the wait-list group on the immediate and 3-month postintervention evaluation of acculturative stress and achieve a higher level of adjustment. Results will have implications for international students, policy makers at universities, the Malaysian Ministry of Higher Education, and future research.

**Trial Registration:**

Clinical Trials Registry India CTRI/2018/01/011223; http://ctri.nic.in/Clinicaltrials/showallp.php?mid1= 21978&amp;EncHid=&amp;userName=Muhamad%20Hanafiah%20Juni

**International Registered Report Identifier (IRRID):**

PRR1-10.2196/12950

## Introduction

### Background

Previous research has identified the challenges and needs of international students during their higher education outside their native countries [1-6]. The most challenging issue faced by international students is adaptation to the new environment, especially the new culture of the host country [7-10]. Other challenges include difficult living circumstances; different foods, climate, cultural norms, and customs [2,8,11-14]; and stressful interactions with local students and the host environments [15]. International students also face academic or educational challenges such as an unfamiliar educational system and language barriers [2,8,11,16]. These stressors can lead to a kind of stress commonly referred as acculturative stress, defined as a psychological and physical discomfort experienced by an individual in a new cultural environment [17].

A causality relationship between a high level of stress experienced by international students and deteriorating health conditions and even more serious health problems has been reported [18,19]. The combination of lack of social relations, financial support, and stress results in students’ feelings of anxiety [5], depression, physical illness, and wasted potential [20-22], leading to depression [23], negatively affecting their academic achievements [24], reducing their academic performance, and even leading them to drop out of university [22,25], especially when it becomes excessive [19]. In this regard, preventing student dropout is a challenging task in higher educational institutions [26]. Pal [27] suggested that programs should be employed in higher educational institutions to address student levels of stress and intention to drop out. Furthermore, research has indicated there is a significant positive relationship between acculturative stress, social support, and sociocultural adjustment of international students [28]. Other studies reported a positive relationship between acculturative stress and psychological adjustment [29,30]. Acculturative stress was found to have a negative relationship with positive acculturation. Acculturative stress is negatively correlated with social support. Consequently, lower levels of acculturative stress are associated with both positive cultural associations and higher levels of social support [31], and acculturation is negatively associated with acculturative stress [32]. In terms of students’ social support and their adjustment, studies on international students in the Malaysian context [32-34] and overseas [28,35] reported that social support significantly affects international students’ adjustment by lowering the level of stress. Similarly, studies have found that social support has positive impacts on sociocultural adaptation as it acts against the stress of cultural adaptation of international students [36,37].

Malaysia has attracted many international students, which has positioned it 11th in world ranking in hosting international students [38]. The increasing number of international students coming from other regions, including the Middle East [39], and the plan to increase intake to 250,000 students by 2025 (Malaysia Education Blueprint 2015-2025: Higher Education) raises the need for enhancing the quality of services and enabling higher educational institutions to meet the needs and requirements of the students, especially in health care. The number of international students has increased from 18,242 to 83,633 in the years 2001-2013, however, making this a challenge. In addition, while the government aims to make the country of Malaysia an international hub for higher education excellence by 2020, research on international postgraduates is still limited. There are only a few studies on international postgraduates that explore the challenges they face in academic and social adjustment in Malaysia [40,41]. Cross-cultural adaptation is crucial for postgraduates in order to overcome the various challenges and become able to psychologically and socioculturally adapt to the new environment [42]. Acculturative stress is caused by stressors such as language barriers, academic barriers, racial discrimination [43,44], perceived surrounding environment, attitudes toward the host country among international postgraduate students [45], cultural shock, homesickness, and perceived hatred [46]. While Rajab et al [46] found that international students’ acculturative stress is moderate, Par et al [47] reported that almost 40% of international postgraduate students joining Malaysian universities experience a high level of acculturative stress, which could affect their academic achievement in higher educational studies [48].

Although the above studies in Malaysia are useful for investigation of international students’ acculturative stress, these studies have focused on describing stress merely from student perception and self-reported reflections, and they are scant and general in comparison with those studies carried out in other host countries [49]. For instance, while research on international student acculturative stress and adaptation has reached its third phase of development in other host countries like Australia [50], in the Malaysian context, such research is still in the early phase of development [49]. In addition, studies in the Malaysian context have ignored the importance of implementation of stress coping interventions. Further empirical investigations into issues pertaining to international student requirements are needed to enable students to improve achievement and performance in higher educational institutions [51]. Based on the above issues, gaps addressed in earlier research, and the need to address stress and adjustment in international students, our study proposes an educational intervention program that can assist international postgraduates to cope with and overcome the different aspects of acculturative stress.

### Theoretical and Conceptual Frameworks

The literature highlights several acculturation models developed by scholars over time, one of which is the bidirectional model of acculturation widely accepted in previous research [52]. Specifically, Berry’s model of acculturation [53] places emphasis on two distinct aspects: the person’s identification with their native culture and identification with the host culture. However, early views and perspectives of acculturation can be valuable in theoretically explaining and discussing the process of acculturation. According to the first view, acculturation is seen as a term covering issues resulting from first-hand contact of individuals with people of different cultural backgrounds. This view also stresses that the consequence of acculturation is changes in the individual’s native or original cultural patterns and behaviors. Based on this view, there is a distinction between acculturation and cultural change in the sense that acculturation encompasses such cultural change as one of its aspects, and assimilation, which is at times a phase of acculturation [54].

The second view emphasizes acculturation as a cultural change initiated by the conjunction of two or more autonomous cultural systems. Thus, acculturative change can result from direct cultural transmission or it can be a result of noncultural aspects, including modification of ecological or demographic characteristics by an impinging culture, and it may be delayed. Hence, acculturation can appear in the form of an internal adaption after the individual accepts alien cultural patterns or behaviors or it can emerge in the individual as a kind of reactive adaptation of traditional modes of life [54,55]. In addition, as defined by Graves [56], psychological acculturation is a process of changes taking place in an individual who participates in a culture-oriented contacting situation directly affected or influenced by the new or external culture and by the changing culture to which they belong. According to Redfield et al [57], acculturation comprises various changes in different forms, identified by Berry [58] as biological, social, and physical changes.

Based on the model of stress developed by Lazarus and Folkman [59], Berry [60] and Berry et al [61] developed an acculturative stress model. The main idea of the framework is that acculturative stress results from situations in which a person’s experiences and appraisal become problematic due to the person’s inability to deal with them and failure to adjust to them through behavioral changes. This implies that acculturative stress is a response of the individual to life events which are grounded in the experience of acculturation [54]. However, not all acculturation changes are assumed to lead to acculturative stress because how acculturation is experienced, perceived, and interpreted by the individual can be influenced by moderating and mediating factors, including personal characteristics such as age, gender, and social support prior and during acculturation [53,62].

In their systematic review of the theoretical frameworks used in research on international students’ sociocultural adaptation to host countries between 2012 and 2017, Sarmiento et al [63] found that 82.2% of the research papers and articles provided a description of particular theoretical frameworks on students’ adaptation. Moreover, 69.3% of these articles reported treating nonspecific frameworks. What was interesting in this review paper was that the majority of the reviewed research papers focused on investigation of international students’ issues related to acculturation and adaption based on the acculturation model. Acculturation should also be approached from the perspective of cross-cultural psychology. As pointed out by Berry [64], acculturation should be investigated in its cultural contexts. In such contexts, researchers can obtain a better understanding of the cultures and individuals in contact. Therefore, such investigation seeks to link the acculturation of a given group of people to which an individual belongs and the individual’s psychological acculturation.

Berry’s [65] acculturation model describes cross-cultural adaptation as a process that involves the individual’s or group’s behavioral and psychological changes in life resulting from their contacts with others from different cultures. Whereas psychological changes are related to one’s modified attitudes, perceptions, and beliefs, behavioral changes are pertinent to their external behavior toward those typical of the host society or mainstream [65]. In a previous study, the term psychological dimension was defined as the person’s perceived acculturative stress which was attributed to their cross-cultural adaptation (eg, how they perceived unfamiliar social cultural customs) [66]. On the other hand, psychological adaptation is often associated with the person’s emotional and affective satisfaction about their integration into a new social environment, while sociocultural adaptation encompasses the process in which the person fits in a new environment and effectively interacts with it [63]. Therefore, our study is framed within the theoretical perspective of acculturation and acculturative stress developed by Berry et al [61] and Berry [53] and further expanded by Berry [65,67,68].

### Study Aims

This study aims to develop, implement, and evaluate the effectiveness of an educational intervention in reducing acculturative stress and improving adjustment among new international postgraduates joining Malaysian public universities. Specifically, the study aims to (1) describe participants’ sociodemographic characteristics, acculturation, acculturative stressors, and social support at the baseline; (2) determine the level of acculturative stress, adjustment (psychological and sociocultural), and intention to drop out of university among new international postgraduates at the baseline; and (3) determine the effect of the educational intervention on acculturative stress, adjustment, and intention to drop out between and within the control and intervention groups at the immediate and 3-month postintervention follow-up.

### Study Hypothesis

In order to achieve the above research objectives, the study attempts to test the following null research hypotheses: (1) the intervention group will score significantly higher than the wait-list group on the immediate and 3-month postintervention evaluation of acculturative stress, adjustment, and intention to drop out measures (between groups) and (2) the acculturative stress, adjustment, and intention to drop out scores for the intervention group at the immediate and 3-month postintervention follow-up will be significantly higher than scores of the baseline or preintervention (within groups).

## Methods

### Overview

The design, conduct, and reporting of the study will adhere to the Standard Protocol Items: Recommendations for Interventional Trials (SPIRIT) [69], Consolidated Standards of Reporting Trials (CONSORT) guidelines [70], and Consolidated Standards of Reporting Trials of Electronic and Mobile Health Applications and Online Telehealth (CONSORT-EHEALTH) [71].

### Study Location

Malaysia is the context of this study since it is the host country for many international students coming from different countries. The target participants in this study will be those new international postgraduate students joining Malaysian public universities. The study includes 20 well-known public universities that attract large numbers of new international students who will be screened according to the inclusion criteria.

### Study Design

**Figure 1:**
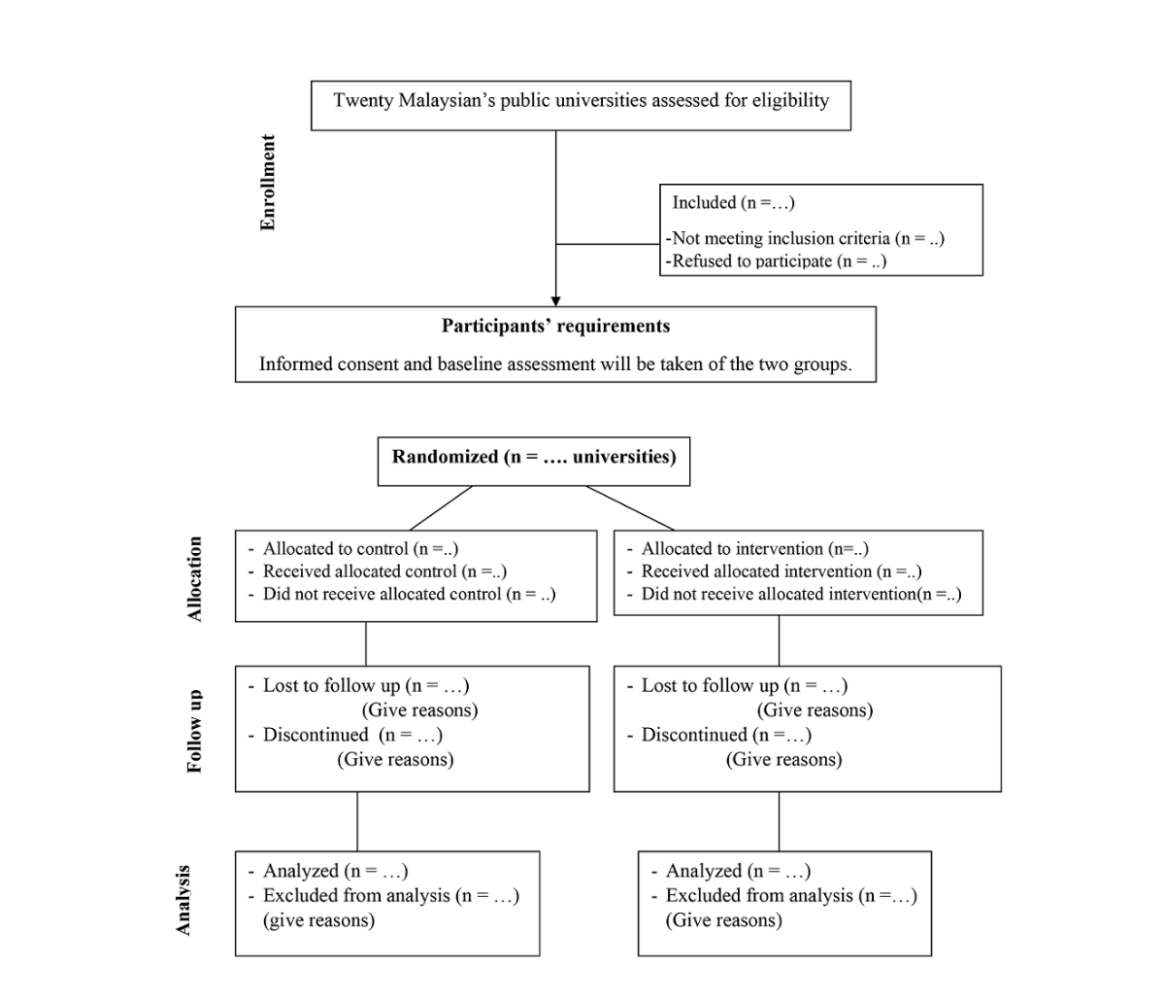
Flowchart of study.

This study is quantitative in nature and experimental in design. Specifically, the study employs a cluster randomized controlled trial (RCT) design. Figure 1 summarizes the study design. Selection of such an experimental design is based on its strength and effectiveness [72]. A cluster design will be employed as the randomization and intervention at the level of the university, and the primary outcome measures are related to students’ acculturative stress, while the secondary outcomes refer to their adjustment and intention to drop out of university. Public universities will be randomized to either a control (wait-list) or intervention (educational intervention) group. In order to determine the effectiveness of the intervention program, the primary and secondary trial outcomes will be assessed through surveys of new international postgraduates at 3 time points: baseline (time 1) before the intervention, immediately postintervention (time 2), and at 3-month follow-up after the intervention (time 3).

### Participants

Public universities that will be eligible to participate in the study should satisfy the following criteria: acting or serving as the main campuses for international students in Malaysia and having more than 40 new international postgraduates in their first semester in Malaysia. For students, only new international postgraduates entering the selected universities in their first academic semester will be eligible to participate.

### Recruitment

For the purpose of eligibility assessment, the researcher will approach all public universities in Malaysia and invite only those universities that are eligible through formal invitation letters issued by the researcher’s university. The letters will be directed to the high authorities of the selected universities through emails seeking their permission for the researcher to conduct his study among international postgraduates. Moreover, the researcher will obtain lists of new international postgraduates joining these universities, including necessary information about the students including contact details, faculty, nationality, and academic program.

Procedures for recruiting public universities will be based on several strategies that have been reported to be useful for maximizing research participation, including prenotifying universities of the study, providing opportunities for participation, visiting persons in charge at the universities, contacting potential participants using telephone and other contact methods, accessing research staff for further clarification on participation, and using dedicated research staff to manage the recruitment process. Specifically, an invitation letter will be directed to a university contact person (deputy vice chancellor for academic and international affairs, deputy vice chancellor for research and innovation, and dean of postgraduate studies) in each eligible university.

Once the letters are sent, the above-mentioned persons in the universities will be contacted by telephone to confirm eligibility based on inclusion criteria and assess interest in participating in the study. This will be followed by several calls until the decision on informing international postgraduates of participation in the study is made and the researcher is informed of such decision. However, such decision, in some cases, will be made through meeting among academic and research staff and consultation with the management committee of a given university. At the end of this process, a confirmation email will be sent to each university, confirming and thanking them for agreement to participate in the study. Each participating university will be given hard and electronic copies of the information and invitation letters for the selected postgraduates in that university.

Student recruitment will use a random sampling procedure to select students from the list of the new postgraduates for each selected university based on probability proportional to size method in order to reduce possible sampling bias. The respondent’s information and consent sheets will be given to the sampled students to get their agreement to participate. In order to maximize response rates, representatives of the international students will be requested to seek the selected postgraduates’ consent.

### Randomization and Blinding

Prior to randomization, the researcher will start collecting baseline data. This will be followed by assigning the universities as the unit of randomization. Universities will be randomized in order to reduce contamination between groups [73,74]. However, to ensure allocation concealment, the allocation sequence of the universities selected at the previous stage will be assigned to a control group and an intervention group by the ratio 1 to 1. The purpose of this is to ensure that the number of universities allocated to each group will remain approximately equal (see Figure 1 flowchart) [75-77]. This will be achieved by employing a simple randomization procedure. The researcher will use a computer-generated list of random numbers. It is exclusive to the researcher who will allocate the 10 universities to the two groups. In addition, no one in the control groups will know what the experimental groups will be offered. The statistician who will perform the primary analyses will be blinded to group allocation. This study will follow a CONSORT chart [73] as shown in Figure 1 (study flowchart).

### Sample Size

**Figure 2:**
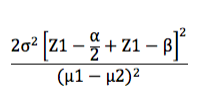
Sample size formula for two population means.

The total sample size needed will be calculated by applying the formula for two population means [78] as seen in Figure 2. Means and standard deviation used for the intervention group and control group were based on the estimation offered in a previous RCT [79]: µ1=2.58, µ2=2.07, SD1=1.08, SD2=1.08, n1=24, n2=30. Therefore, the total sample (n) will account for 71 participants. Calculation of the required sample size in this study was performed based on the guidelines in the CONSORT for cluster RCTs [73]. By considering the account intraclass correlation coefficient (ICC), it is necessary to multiply the sample size in RCTs by design effect=1+(m–1)*ICC, where m is the average cluster size [80-83]. Thus, the average class size is assumed to be 40 participating students, and an ICC of .05 could be expected. This would result in design effect N=1+(m–1)*ICC=1+(40–1)*.05=2.95. Based on the sample size required by the formula, the required minimum sample size for each group after adjusting for design effect is n=71*2.95=209, and the total sample size required is 209 participants per arm.

To factor in 20% attrition, if (n) is the sample size required as per formula and (d) is the dropout rate, adjusted sample size N new is obtained as N1=n/(1–d) [84]. Therefore, the total required per group after adjusted for dropout rate is N1=209/(1–0.2)=261; hence, a total of 522 new international postgraduate students will be needed for the sample size in both groups.

### Intervention

#### Intervention Development

The intervention is developed based on the acculturation and acculturative stress models integrating with a cognitive behavioral theory with the aim of focusing on cross-cultural sessions and social and behavioral issues in order to improve social and psychological adjustment and reduce acculturative stress and intention to drop out among international students, especially those who have just arrived at the new hosting environment. Most of these models highlight important variables, including acculturation experience, acculturation strategies, and social support, which have an effect on decreasing acculturative stress and a positive impact on the adjustment of students in host cultures [68,85,86]. Therefore, this study developed an intervention that targets individual variables such as acculturation, social support, and coping strategies, as these factors have the potential to mitigate acculturative stress and, consequently, the international students’ adjustment and intention to drop out.

The educational intervention was developed based on previous experimental studies on the implementation of intervention programs such as Excellence in Experiential Learning and Leadership developed by Mak et al [87], a cultural transition course for international students by Brunsting et al [88], a behavioral intervention to improve cross-cultural relationships consistent with the acculturation models developed by Pritchard and Skinner [89], a cognitive behavioral group intervention on acculturation by Pan et al [90], a group psychological intervention to enhance coping and acculturating by Smith and Khawaja [91], and further intervention and support programs aiming to improve international students’ sociocultural and psychological adjustment and reduce their acculturative stress and intention to drop out of the university [92-94]. Taking into consideration the limitations of these previous intervention studies, development of the intervention in this study is based on the creative use of cross-cultural activities in a program, using a clear quantitative evaluation of the effectiveness of the program and including components that directly target international students’ psychological and sociocultural adjustment and acculturative stress. The intervention was also developed based on the results of previous studies.

Berry’s model [68] for acculturation places an emphasis on individuals’ cognitive appraisal of acculturative life changes as another factor that has an impact on acculturation. For instance, a given person is likely to appraise a life change as a chance or, alternately, as a stressor that is conducive to acculturative stress. Berry’s model provides a clear picture of the instance or case in which a person appraises a life change as a stressor. Moreover, they may make an additional appraisal of the sufficient coping resources that they have in order to overcome the stressor. Thus, acculturative stress will become low when a person possesses adequate coping resources. As a result, they will be able to psychologically and socioculturally adjust to the new host country. Therefore, the model adopted in this study guides development of the intervention (identifying the necessary components of what an efficient intervention that reduces acculturative stress and improves adjustment should be). These are cognitive appraisals of acculturative life changes, acculturation experience, stressors, acculturative stress, and coping strategies including social support [95]. To achieve this, the study adopts a cognitive behavioral framework for acculturation and acculturative stress since it suits the purpose of this effective intervention. The framework addresses cognitive appraisals and increases coping resources in the cognitive and behavioral domains.

#### Intervention Content

The proposed program will cover a period of 7 sessions and will be conducted over a period of 9 hours in each university of the intervention group. Core modules of the Acculturative Stress Educational Intervention Program will include (1) introduction to the intervention program, (2) introduction to Malaysia and Malaysian culture and customs, (3) study abroad in Malaysia, (4) understanding acculturation, (5) acculturative stress, (6) coping strategies, and (7) adjustment strategies. In each session, participating international students will be offered different topics with experiential and interactive methods of delivery and materials. Websites; videos; demonstrations; in-group activities and interaction; interchanging of cultural background, experience, and intracultural communication; and critical incidents with analysis, reflection, and discussion on different cases and scenarios of international students’ experience with acculturative stress and adjustment will be used for enabling students to engage with the program.

#### Intervention Implementation

The aim of the intervention program is to transfer knowledge and gain skills, and the program will cover two parts: (1) a seminar session with PowerPoint (Microsoft Corp) presentation covering the 7 sessions, providing knowledge about different aspects of Malaysia and Malaysian culture, acculturation, and acculturation strategies and (2) knowledge and activities will be provided about acculturative stress, the sources for acculturative stress, adjustment, coping strategies, and social support. In this part, group exercises and activities will be used for enabling the students to engage with the program and gain knowledge and skills. Therefore, the intervention program will be delivered through seminar sessions, a PowerPoint presentation, videos, group activities, a printed booklet, and other printed materials that are available on the website during the intervention and study but can be accessed only by the intervention group using codes. Website, email, and WhatsApp are the primary methods to deliver the educational materials to the intervention group. These technological tools will be used to send short reminder messages and encourage participants to take part in the intervention and control groups. Participants in the study will be provided useful information about the topic and how to cope and adjust in the new environment. The second phase focuses on participants’ independent processing and implementing of information provided in the first phase as they begin to use the information in their personal lives and develop the skills necessary for enhancing their situation.

### Study Variables and Instruments

This study will use a self-administered questionnaire consisting of 8 sections measuring the sociodemographic, independent, and dependent variables (study outcomes). The questionnaires will be completed at time 1, time 2, and time 3.

#### Primary Outcome: Acculturative Stress

Stress is defined as a state of a person that results from their interaction with their surroundings and is regarded as a threat to the well-being of the individual. Since this study focuses on international students’ acculturative stress as the primary outcome, we conceptualize acculturative stress as stress that a person suffers from reaction or response to stressful events of life relevant to the process of acculturation [65] as well as the psychological difficulties in the new culture [96]. Acculturative stress is also a psychosocial result of the individual’s lacking familiarity with the customs and social norms of the host culture [97,98]. Hence, one of the focuses of the investigation in this study is the acculturative stress experienced by international students in the Malaysian context [99-101].

This study uses the Acculturative Stress Scale for International Students (ASSIS) for measuring the primary outcome, international students’ acculturative stress. The measure is effective in examining international students’ cross-cultural adjustment, specifically the extent to which they find their involvement in everyday social situations as difficult due to cultural differences. ASSIS is widely used in studies on international students’ acculturative stress in Malaysia, and the scale is also highly reliable and valid as reported in early research [29,32,66,101-106]. The overall score for the ASSIS is in the range of 36 to 180. The use of the total score of the ASSIS is highly encouraged by Sandhu and Asrabadi [103]. In the case of using the total scores, high scores mean that the individual’s perceived acculturative stress is high [46,104-106].

#### Secondary Outcomes: Adjustment and Intention to Drop Out

Adjustment is known as a short-term and dynamic process experienced by the individual in relation to the new cultural environment; it also refers to the degree to which a certain university meets the demands or requirements of international students [107-109]. According to Ward and Kennedy [110], there are two dimensions of adjustment: sociocultural and psychological.

First, sociocultural adjustment is a process in which individuals with different cultural backgrounds become skilled and able to engage in negotiating the host culture and effectively interacting with its local people [111]. Assessment of the individual’s sociocultural adaptation is based on a 20-item version of the Sociocultural Adaptation Scale (SCAS) [110]. Participants will be asked questions regarding the degree of difficulty experienced by them in a number of areas [110,112-115]. This is based on a 5-point Likert scale for rating the difficulty of 20 daily situations adapted by the participating international students in the Malaysian context. Initial use of the SCAS was documented in a previous study that indicated it was reliable, with scale alphas ranging from .75 to .91, indicating good internal consistency and construct validity [110-115]. The SCAS is regarded as a flexible research instrument and measure that is easily modified based on characteristics of the sojourning sample, and it can be adapted to different cultural contexts [34].

Second, the Satisfaction with Life Scale (SWLS) is used to measure psychological adjustment and the cognitive component of subjective well-being [116] through 5 items. Consistent with the standpoint of the World Health Organization on health, psychological adjustment is seen as state of well-being and not merely the absence of disease [117]. Hence, its specific focus is on evaluating individuals’ perceived satisfaction with life as a whole based on their criteria and views [118,119]. The items on the SWLS are based on a 5-point scale on which respondents are asked to agree or disagree with 5 statements (eg, “In most ways my life is close to my ideal”). The scale is available in a range of languages and has been used in cross-cultural research [118], and its internal consistency for psychological adjustment has proved to be very good [34,42,109,119].

Finally, the international students’ intention to drop out of the university is also a secondary outcome. This variable describes the international students’ intended behavior concerning their persistence of studies in the host country. It will be measured using this single item: How likely is it that you will withdraw from university (for whatever reason)? This question underlying the dependent variable of students’ intention to drop out is known to be the best predictor of students’ actual behavior and can be used in the absence of behavioral data [120,121]. In our study, this question is intended to seek participating students’ responses to whether they were thinking of or had already thought of dropping out of university.

#### Sociodemographic Information Questionnaire

All participants will be asked to provide sociodemographic information including their gender, age, marital status, educational level, duration of stay in Malaysia, country of origin, prior traveling experience, native language, and self-reported proficiency of language. Self-reported proficiency will also be assessed using a composite score from the following questions: (1) What is your present level of English fluency? (2) How comfortable are you communicating in English? (3) How often do you communicate in English? This method of measuring and assessing English language proficiency has been documented previously by several researchers [122-125]. For this study, participants will be asked to rate their perceived proficiency in speaking, understanding, writing, and having a conversation in Malay and English, as language skills are said to enable sojourners to interact with host nationals and engage in interpersonal relationships, which, in turn, influences their adaptation [125,126].

#### Other Independent Variables and Measurements

This study focuses on other independent variables that directly or indirect affect study outcomes: acculturation, social support, and acculturative stressors.

Acculturation is a process of adaptation or adjustment to a new cultural context that imposes behavioral, cultural, and psychological changes on the individual in an attempt to be in contact with other individuals or even groups coming from diverse cultural backgrounds [65,127]. Participants’ acculturation will be measured using the Acculturation Index (AI) [128]. The AI was adopted from the two-dimensional acculturation model of Berry et al [61]. These two basic dimensions of acculturation are students’ identification with their heritage culture and their relationship with the host culture. The scale comprises 21 cognitive and behavioral items (eg, food, language, recreational activities, social customs, pace of life, religious beliefs). The instrument is intended to identify acculturation strategies used by participants [110,115,128,129]. Participants will be given two questions on their current lifestyles in two cultures: “How similar are your experiences and behaviors to members of your culture of origin?” and “How similar are your experiences and behaviors to members of Malaysian culture?” This instrument makes use of a 5-point, partly anchored, Likert-type scale ranging from 1=not at all similar to 5=very similar. Independent scores for the two dimensions are in the range of 0 and 105, and higher scores are indicative of the participants’ stronger identification with the host culture. In previous studies, internal consistencies and reliabilities were illustrated by Cronbach alphas ranging from .91 to .94 and .89 to .97 for the heritage culture and host culture, respectively. These reliabilities were confirmed by studies using this measure among sojourns from different cultures living in different populations [115,128,129].

While acculturative stressors are those related to international students’ acculturative process at the biological, social, functional, cultural, and physical/environmental levels [130], other related stressors include reentry issues. Acculturative stressors will be measured by the International Student Acculturative Stressor Scale (ISASS) developed by Eustace [131] for the purpose of capturing the degree to which each acculturative stressor represents an issue or difficulty for the international students participating in this study. Respondents will be provided with a 5-point Likert-type scale with 13 items that allow them to rate their degree of difficulty of the different acculturative stressors. The options ranged from 1=not difficult at all to 5=very difficult. The total scores for the 13 items ranged between 13 and 65. Higher scores indicate higher levels of difficulty of such acculturative stressors.

Perceived social support is another dependent variable, defined as the individual’s impression of the available resources that are supportive for them during their experience of stress and symptoms experienced. Social support encompasses three different dimensions: family, friends, and significant others. Whereas family and friends are recognized as self-explanatory, significant others might be a supervisor, peer, colleague, or any other person with whom the stressed person has constant contact [42,132,133]. In this study, the Multidimensional Scale of Perceived Social Support (MSPSS) aims to measure international students’ perceived social support. It was designed and developed by Zimet et al [132] based on adult samples. Several studies have used the MSPSS with the aim of measuring perceived social support across different cultures [119,132,134-136]. It is reported to be a short, clear, and accurate scale that can be used for measuring social support. The MSPSS is also relatively free of the biases of social desirability [137]. Using this scale, respondents select from the 5-point Likert-type scale choices ranging from 1=strongly disagree to 5=strongly agree. In a previous study, the factor loadings of these items were relatively high, and the measure had internal consistencies (Cronbach alphas) of .79, .81, and .82 for family, friend, and significant other support, respectively. In another study in the Malaysian context, internal consistency results were reported of .88, .64, and .87 for family support, friend support, and the availability of a special person [42,109,119].

### Quality Assessment

Prior to distributing the questionnaires to the targets of the study, the researcher conducted a pilot study as recommended by scholars to reduce ambiguity, validate the instrument, and evaluate the educational intervention program before conducting the study [138,139]. For the validation of the intervention module and educational materials, the researcher constructed the basis of each statement, information, guideline, or strategy in the educational module from credible published scientific sources. A panel of experts then engaged in reviewing all sources and the newly developed educational intervention before providing it to the study sample. The experts read the references used in developing the module and educational materials and were asked to provide their evaluation and suggestions for enhancement. This was followed by resending the final module to the panel of experts for a second review. This resulted into the final version of the educational module and educational materials that will be used in this study.

After the educational module and materials were designed, a group of experts in psychiatry and education (3 experts from the psychiatric department, faculty of medicine, and 3 from the faculty of education) rated the content validity of the educational module and materials from the proposed educational intervention in terms of its accuracy, currency, and appropriateness of content using a 5-point scale: 1=extremely unsuitable to 5=extremely suitable. They will be also asked to identify areas of items that need to be improved, removed, or modified [140]; we will then pretest the educational module and materials among new international students in a private university to evaluate the reliability of the educational module and materials.

### Statistical Analysis

We will use SPSS Statistics version 25.0 (IBM Corp) to analyze the data. Statistical significance will be considered as a *P* value of less than .05. Analysis of the data will take into account clustering of students within universities. The study will use statistical analyses, including descriptive statistics of the respondents’ sociodemographic characteristics, chi-square test for nonparametric variables, and 2-tailed *t* test for continuous variables, to compare between the control and intervention groups on sociodemographic variables and primary and secondary outcome measures. Complete case analyses, intent-to-treat analyses, and completer status analyses will be conducted according to the initial allocation of universities to either intervention or control groups. In relation to the missing data, we will follow the guidelines for analyzing and reporting cluster RCTs with missing data established by Fiero et al [141] and Díaz et al [142].

The effectiveness of the intervention will be assessed using the generalized estimation equation (GEE). Specifically, the differences in the research hypothesis in terms of students’ acculturative stress, adjustment (sociocultural and phycological), and intention to drop out will be compared at 3 points—time 1 (preintervention), time 2 (immediately postintervention), and time 3 (3-month follow-up)—between and within the intervention and the control groups adjusted for covariate variables. The GEE method was chosen because of its efficiency in appropriately modeling the structure of the correlations of the pre-post repeated measures and its low degree of reliance on the assumption of normality in the distributions of data for the variables in the analysis. Furthermore, in the context of RCT research, multilevel models such as the GEE are the most appropriate model because they permit the estimation of treatment effects (ie, group differences) across multiple time points within a single statistical model [143].

However, there is no default option in the GEE analysis for eta squared to obtain the intervention effect size that can be expressed as a standardized effect size. Therefore, two popular measures, Cohen d and eta squared, will be used in this study. Cohen d = beta ÷ standard deviation, with beta representing the difference between two means for the two groups relative to their standard deviation obtained from the pooled variance to compute the effect size within group and between group [144-146]. Hence, d = (mean 1 – mean 2) / (the average standard deviation of the two groups). The eta squared indicates how much of the total variance is explained by the difference between the means. Therefore, we will convert the effect size d to eta squared with the equation η^2^=d^2^/d^2+4^. The reported effect size d is also interpreted by Cohen [147] as adopted by many previous studies, specifically intervention as well in terms of the practical or clinical significance of the effect, 0.2 (small effect), 0.5 (moderate effect), and 0.8 (large effect), and similarly for eta squared 0.01 (small effect), 0.06 (moderate effect), and 0.14 (large effect) [146,148-151].

## Results

The trial protocol of this study was approved by the Universiti Putra Malaysia Ethics Committee for Research Involving Human Subjects (reference: JKEUPM [FPSK-P104] 2017). The trial was registered at Clinical Trials Registry India [CTRI/2018/01/011223]. Approval for participation in the study was obtained from the authorities of the selected public universities. The study results will be reported at the cluster and individual levels, including information about the level of acculturative stress, adjustment, intention to drop out of the university, effectiveness of the intervention, estimated effect size and its precision, and ICCs for each primary and secondary outcome. Initial results are expected to be submitted for publication by the end of the first semester 2019/2020, and the paper will be presented at national and international conferences.

## Discussion

### Summary

This study investigates acculturative stress, adjustment, and intention to drop out among new international postgraduates joining Malaysian public universities. Specifically, the focus of the study is on the effectiveness of a proposed educational intervention in reducing new international postgraduates’ acculturative stress in the host country of Malaysia. Due to the fact that Malaysia is becoming one of the important host countries for postgraduates in the Asian region, it is important to investigate acculturative stress and adaptation of new international postgraduates to the culture and environment. The study is expected to contribute to previous studies and existing knowledge about the challenges and barriers faced by postgraduates entering higher education in Malaysia. As an experimental study, the results will inform us of the stressors that cause acculturative stress and strategies that can be used by international postgraduates to adapt to the new environment. Another contribution of this study is the implemented intervention program that can be used as a guide for further studies among new international students. These results will provide good insight into this important research topic and be of value for international postgraduates, academics, authorities, policy makers in higher education, and researchers interested in exploring issues and challenges among postgraduates in different higher educational contexts.

### Conclusions

This trial is expected to provide valuable insight into the implementation and effectiveness of educational intervention programs in reducing new international postgraduates’ acculturative stress and improving their adjustment to the host culture. The aim of designing the initial cluster RCT is also to provide information regarding the feasibility of potential future full-scale trials in order to optimize the intervention and design approach. Process evaluation outcomes will provide contextual information about implementation decisions of international students, researchers, and higher education stakeholders.

## Abbreviations

AI: Acculturation Index
ASSIS: Acculturative Stress Scale for International Students
CONSORT: Consolidated Standards of Reporting Trials
CONSORT-EHEALTH: Consolidated Standards of Reporting Trials of Electronic and Mobile Health Applications and Online Telehealth
GEE: generalized estimation equation
ICC: intraclass correlation coefficient
ISASS: International Student Acculturative Stressor Scale
MSPSS: Multidimensional Scale of Perceived Social Support
RCT: randomized controlled trial
SCAS: Sociocultural Adaptation Scale
SPIRIT: Standard Protocol Items: Recommendations for Interventional Trials
SWLS: Satisfaction with Life Scale

## References

[1] Alavi M, Mansor SMS (2011). Categories of problems among international students in Universiti Teknologi Malaysia. Procedia Soc Behav Sci.

[2] Andrade MS (2016). International students in English-speaking universities. J Res Int Educ.

[3] Arthur N (2003). Counseling International Students: Clients From Around the World.

[4] Gebhard (2012). International students' adjustment problems and behaviors. J Int Students.

[5] Poyrazli S, Grahame K (2007). Barriers to adjustment: needs of international students within a semi-urban campus community. J Instruct Psychol.

[6] Leon RA, Chmiel J (2013). Counseling international students: clients from around the world. J Int Students.

[7] Guidry Lacina J (2002). Preparing international students for a successful social experience in higher education. New Directions Higher Educ.

[8] Mahmud Z, Amat S, Rahman S, Ishak NM (2010). Challenges for international students in Malaysia: culture, climate and care. Procedia Soc Behav Sci.

[9] Hyun J, Quinn B, Madon T, Lustig S (2007). Mental health need, awareness, and use of counseling services among international graduate students. J Am Coll Health.

[10] Osman F, Salari R, Klingberg-Allvin M, Schön U, Flacking R (2017). Effects of a culturally tailored parenting support programme in Somali-born parents’ mental health and sense of competence in parenting: a randomised controlled trial. BMJ Open.

[11] Bektaş DY (2008). Counselling international students in Turkish universities: current status and recommendations. Int J Adv Counselling.

[12] Shatkin J (2007). Transition to college: separation and change for parents and students.

[13] Barletta J, Kobayashi Y (2012). Cross-cultural counselling with international students. Aust J Guid Couns.

[14] Mori S (2000). Addressing the mental health concerns of international students. J Couns Devel.

[15] Pandian A (2002). English language teaching in Malaysia today. Asia Pacific J Educ.

[16] Furnham A (2004). Foreign students: education and culture shock. Psychologist.

[17] Lee DS, Padilla AM (2014). Acculturative stress and coping: gender differences among Korean and Korean American university students. J Coll Student Devel.

[18] Gupchup G, Borrego M, Konduri N (2004). The impact of student life stress on health related quality of life among doctor of pharmacy students. Coll Student J.

[19] Shields N (2001). Stress, active coping, and academic performance among persisting and nonpersisting college students. J Appl Biobehav Res.

[20] Walton R (2002). A Comparison of Perceived Stress Levels and Coping Styles of Junior and Senior Students in Nursing and Social Work Programs [Dissertation].

[21] Misra R, Crist M, Burant CJ (2003). Relationships among life stress, social support, academic stressors, and reactions to stressors of international students in the United States. Int J Stress Manag.

[22] Pereira A (1997). Helping Students Cope: Peer Counselling in Higher Education [Dissertaion].

[23] Sulaiman SMA (2013). Emotional intelligence, depression and psychological adjustment among university students in the Sultanate of Oman. Int J Psychol Studies.

[24] Whitman N (1985). Student stress: effects and solutions. ERIC Digest 85-1.

[25] Misra R, McKean M, West S, Russo T (2000). Academic stress of college students: Comparison of student and faculty perceptions. College Student Journal.

[26] Tinto V (2016). Research and practice of student retention: what next?. J Coll Student Retention Res Theory Pract.

[27] Pal S (2012). Mining educational data to reduce dropout rates of engineering students. IJIEEB.

[28] Han SJ, Han KH (2016). A research on the stress of cultural adaptation and adaptation to the Korean culture of the international students from Mongolia. Int Inform Inst (Tokyo).

[29] Yeh CJ, Inose M (2003). International students' reported English fluency, social support satisfaction, and social connectedness as predictors of acculturative stress. Couns Psychol Q.

[30] Sovic S (2008). Coping with stress: the perspective of international students. Art Design Commun Higher Educ.

[31] Sullivan C (2010). Predictors of Acculturative Stress for International Students in the United States [Dissertation].

[32] Falavarjani MF, Yeh CJ, Brouwers SA (2019). Exploring the effects of acculturative stress and social support on the acculturation-depression relationship in two countries of similar social status. Int Migrat Integr.

[33] Lashari S, Kaur A, Awang-Hashim R (2018). Home away from home—the role of social support for international students’ adjustment. Malay J Learn Instruc.

[34] Freeman K, Nga E, Mathews M (2017). International students challenges and academic adjustment in higher education in Malaysia. Sci Int (Lahore).

[35] Lee J, Koeske GF, Sales E (2004). Social support buffering of acculturative stress: a study of mental health symptoms among Korean international students. Int J Intercult Relat.

[36] Kim E (2007). Acculturative Stress, Social Support and Social Adjustment of Marriage-Based Immigrant Women [Thesis].

[37] Cohen S, Wills TA (1985). Stress, social support, and the buffering hypothesis. Psychol Bull.

[38] Cheng MY, Mahmood A, Yeap PF (2013). Malaysia as a regional education hub: a demand-side analysis. J Higher Educ Policy Manag.

[39] Talebloo B, Baki R (2013). Challenges faced by international postgraduate students during their first year of studies. Int J Human Soc Sci.

[40] Singh JKN, Jack G (2017). The benefits of overseas study for international postgraduate students in Malaysia. High Educ.

[41] Singh J, Schapper J, Jack G (2014). The importance of place for international students? choice of university A case study at a malaysian university. Journal of Studies in International Education.

[42] Shafaei A, Razak NA (2016). What matters most: importance-performance matrix analysis of the factors influencing international postgraduate students’ psychological and sociocultural adaptations. Qual Quant.

[43] Nursyazana M (2011). The Sources of Acculturative Stress Among International Students [Thesis].

[44] Shafaei A, Abd Razak N, Nejati M (2016). Integrating two cultures successfully: Factors influencing acculturation attitude of international postgraduate students in Malaysia. Jnl of Research in Internatl Education.

[45] Desa A, Yusooff F, Kadir NBA (2012). Acculturative stress among international postgraduate students at UKM. Procedia Soc Behav Sci.

[46] Rajab A, Rahman H, Panatik S, Mansor N (2014). Acculturative stress among international students. Journal of Economics, Business and Managemen.

[47] Par M, Hassan SA, Uba I, Baba M (2015). Perceived stress among international postgraduate students in Malaysia. IJPS.

[48] Puspitasari A (2011). The Influence of Perceived Stress and Culture Shock Among International Postgraduate Students' Academic Achievement in Universiti Utara Malaysia [Thesis].

[49] Saad NSM, Yunus MM, Embi MA (2013). Research on international students in traditional host countries and Malaysia: some potential areas in Malaysia. Procedia Soc Behav Sci.

[50] Dawson J, Hacket J (2006). Developing service quality for international students. www.idp.com/aiec.

[51] Saleh H, Qasem A (2010). International and local students' satisfaction of healthcare services. J Bus Manag Acct.

[52] Cuellar I, Arnold B, Maldonado R (2016). Acculturation rating scale for Mexican Americans II: a revision of the original ARSMA scale. Hisp J Behav Sci.

[53] Berry JW (1997). Immigration, acculturation, and adaptation. Appl Psychol.

[54] Kuo BC (2014). Coping, acculturation, and psychological adaptation among migrants: a theoretical and empirical review and synthesis of the literature. Health Psychol Behav Med.

[55] Barnett Hg (1954). Acculturation: an exploratory formulation—the Social Science Research Council Summer Seminar on Acculturation, 1953. Am Anthropol.

[56] Graves TD (1967). Psychological acculturation in a tri-ethnic community. Southwestern J Anthropol.

[57] Redfield R, Linton R, Herskovits MJ (1936). Memorandum for the study of acculturation. Am Anthropol.

[58] Berry J (1983). Acculturation as varieties of adaptation. Acculturation: Theory, Models, And Some New Findings.

[59] Lazarus RS, Folkman S, Gentry WD (1984). Coping and adaption. The Handbook of Behavioral Medicine.

[60] Berry J (2006). Acculturative stress. Handbook of Multicultural Perspectives on Stress and Coping.

[61] Berry JW, Kim U, Minde T, Mok D (1987). Comparative studies of acculturative stress. Int Migr Rev.

[62] Sam D, Berry J (2006). The Cambridge Handbook of Acculturation Psychology.

[63] Sarmiento AV, Pérez MV, Bustos C, Hidalgo JP, del Solar JIV (2019). Inclusion profile of theoretical frameworks on the study of sociocultural adaptation of international university students. Int J Intercult Relat.

[64] Berry J (2002). Cross-Cultural Psychology: Research and Applications.

[65] Berry JW (2005). Acculturation: living successfully in two cultures. Int J Intercult Relat.

[66] Zhang Y, Garcia-Murillo MA (2018). Improving international students' cultural skills through a school-based program. Int Res Rev.

[67] Berry J (2003). Conceptual Approaches to Acculturation.

[68] Berry J (2006). Stress Perspectives on Acculturation.

[69] Chan A, Tetzlaff J, Altman D, Laupacis A, Gøtzsche P (2013). SPIRIT statement: defining standard protocol items for clinical trials. Ann Internal Med.

[70] Moher D, Hopewell S, Schulz KF, Montori V, Gotzsche PC, Devereaux PJ, Elbourne D, Egger M, Altman DG (2010). CONSORT 2010 Explanation and Elaboration: updated guidelines for reporting parallel group randomised trials. BMJ.

[71] Eysenbach G (2011). CONSORT-EHEALTH: Improving and standardizing evaluation reports of web-based and mobile health interventions. J Med Internet Res.

[72] Creswell J (2013). Qualitative, Quantitative, and Mixed Methods Approaches.

[73] Campbell MK, Piaggio G, Elbourne DR, Altman DG (2012). Consort 2010 statement: extension to cluster randomised trials. BMJ.

[74] Eccles M, Grimshaw J, Campbell M, Ramsay C (2003). Research designs for studies evaluating the effectiveness of change and improvement strategies. Qual Safety Health Care.

[75] Efird J (2010). Blocked randomization with randomly selected block sizes. Int J Envir Res Public Health.

[76] Suresh K (2011). An overview of randomization techniques: an unbiased assessment of outcome in clinical research. J Hum Reprod Sci.

[77] Altman DG, Bland JM (1999). How to randomise. BMJ.

[78] Ogston SA, Lemeshow S, Hosmer DW, Klar J, Lwanga SK (1991). Adequacy of sample size in health studies. Biometrics.

[79] Tavakoli S, Lumley MA, Hijazi AM, Slavin-Spenny OM, Parris GP (2009). Effects of assertiveness training and expressive writing on acculturative stress in international students: a randomized trial. J Couns Psychol.

[80] Campbell MK, Elbourne DR, Altman DG (2004). CONSORT statement: extension to cluster randomised trials. BMJ.

[81] Gao F, Earnest A, Matchar DB, Campbell MJ, Machin D (2015). Sample size calculations for the design of cluster randomized trials: a summary of methodology. Contemp Clin Trials.

[82] van Breukelen GJ, Candel MJ (2012). Calculating sample sizes for cluster randomized trials: we can keep it simple and efficient!. J Clin Epidemiol.

[83] García-Escalera J, Valiente RM, Chorot P, Ehrenreich-May J, Kennedy SM, Sandín B (2017). The Spanish version of the unified protocol for transdiagnostic treatment of emotional disorders in adolescents (UP-A) adapted as a school-based anxiety and depression prevention program: study protocol for a cluster randomized controlled trial. JMIR Res Protoc.

[84] Suresh K, Chandrashekara S (2012). Sample size estimation and power analysis for clinical research studies. J Hum Reprod Sci.

[85] Arends-Tóth J, van de Vijver FJ, Bornstein M, Cote L (2006). Issues in the conceptualization and assessment of acculturation. Acculturation and Parent-Child Relationships: Measurement and Development.

[86] Safdar S, Struthers W, van Oudenhoven JP (2009). Acculturation of Iranians in the United States, the United Kingdom, and the Netherlands. J Cross-Cult Psychol.

[87] Mak A, Westwood M, Barker M, Ishiyama I (1998). Developing sociocultural competencies for success among international students: the ExcelL programme. J Int Educ.

[88] Brunsting NC, Smith AC, Zachry CE (2018). An academic and cultural transition course for international students: efficacy and socioemotional outcomes. J Int Students.

[89] Pritchard RMO, Skinner B (2016). Cross-cultural partnerships between home and international students. J Stud Int Educ.

[90] Pan J, Ng P, Young DK, Caroline S (2016). Effectiveness of cognitive behavioral group intervention on acculturation. Res Soc Work Pract.

[91] Smith RA, Khawaja NG (2015). A group psychological intervention to enhance the coping and acculturation of international students. Adv Mental Health.

[92] Carr JL, Miki Koyama M, Thiagarajan M (2003). A women's support group for Asian international students. J Am Coll Health.

[93] Binder N, Schreier M, Kühnen U, Kedzior K (2013). Integrating international students into tertiary education using intercultural peer-to-peer training at Jacobs University Bremen, Germany. J Educ Train Stud.

[94] Abe J, Talbot D, Gellhoed R (1998). Effects of a peer program on international student adjustment. J Coll Student Devel.

[95] Poyrazli S, Kavanaugh P, Baker A, Al-Timimi N (2004). Social support and demographic correlates of acculturative stress in international students. J Coll Couns.

[96] Smart JF, Smart DW (2016). Acculturative stress. Couns Psychol.

[97] Lin J, Yi J (1997). Asian international students' adjustment: issues and program suggestions. Coll Student J.

[98] Ahrari S, Krauss S, Suandi T, Abdullah HB, Sahima A, Olutokunbo A, Dahalan D (2019). A stranger in a strange land: experiences of adjustment among international postgraduate students in Malaysia. Issues Educ Res.

[99] Ye H, Juni MH (2017). Acculturative stress level among international postgraduate students of a public university in Malaysia. Int J Public Health Clin Sci.

[100] Akhtar M, Kröner-Herwig B (2015). Acculturative stress among international students in context of socio-demographic variables and coping styles. Curr Psychol.

[101] Falavarjani MF, Yeh CJ (2017). The impact of acculturation identification and acculturative stress on creativity among Iranian immigrants living in Malaysia. J Ethnic Migr Stud.

[102] Franco M, Hsiao Y, Gnilka PB, Ashby JS (2018). Acculturative stress, social support, and career outcome expectations among international students. Int J Educ Vocat Guidance.

[103] Sandhu DS, Asrabadi BR (2016). Development of an acculturative stress scale for international students: preliminary findings. Psychol Rep.

[104] Constantine MG, Okazaki S, Utsey SO (2004). Self-concealment, social self-efficacy, acculturative stress, and depression in African, Asian, and Latin American international college students. Am J Orthopsych.

[105] Poyrazli S, Thukral R, Duru E (2010). International students' race-ethnicity, personality and acculturative stress. J Psychol Couns.

[106] Wei M, Heppner PP, Mallen MJ, Ku T, Liao KY, Wu T (2007). Acculturative stress, perfectionism, years in the United States, and depression among Chinese international students. J Couns Psychol.

[107] Feldt RC, Graham M, Dew D (2017). Measuring adjustment to college: construct validity of the student adaptation to college questionnaire. Measure Eval Couns Devel.

[108] Mesidor J, Sly K (2016). Factors that contribute to the adjustment of international students. J Int Students.

[109] Shafaei A, Razak NA (2016). International postgraduate students’ cross-cultural adaptation in Malaysia: antecedents and outcomes. Res High Educ.

[110] Ward C, Kennedy A (1999). The measurement of sociocultural adaptation. Int J Intercult Relat.

[111] Searle W, Ward C (1990). The prediction of psychological and sociocultural adjustment during cross-cultural transitions. Int J Intercult Relat.

[112] Ward C, Kennedy A (1993). Psychological and socio-cultural adjustment during cross-cultural transitions: a comparison of secondary students overseas and at home. Int J Psychol.

[113] Zung WWK (1965). A self-rating depression scale. Arch Gen Psychiatry.

[114] Ward C, Kennedy A, Pandey J, Sinha D, Bhawuk D (1996). Crossing cultures: the relationship between psychological and sociocultural dimensions of cross-cultural adjustment. Asian Contributions to Cross-Cultural Psychology.

[115] Ward C, Rana-Deuba A (2016). Acculturation and adaptation revisited. J Cross-Cult Psychol.

[116] Diener E, Emmons RA, Larsen RJ, Griffin S (2010). The satisfaction with life scale. J Personality Assess.

[117] Sam DL (2000). Psychological adaptation of adolescents with immigrant backgrounds. J Soc Psychol.

[118] Pavot W, Diener E (1993). Review of the satisfaction with life scale. Psychol Assess.

[119] Yusoff Y, Othman A (2011). An early study on perceived social support and psychological adjustment among international students: the case of a higher learning institution in Malaysia. Int J Bus Soc.

[120] Lounsbury JW, Saudargas RA, Gibson LW (2004). An investigation of personality traits in relation to intention to withdraw from college. J Coll Student Devel.

[121] Harris P, Campbell Casey S, Westbury T, Florida-James G (2015). Assessing the link between stress and retention and the existence of barriers to support service use within higher education. J Further Higher Educ.

[122] Meng Q, Zhu C, Cao C (2017). Chinese international students’ social connectedness, social and academic adaptation: the mediating role of global competence. High Educ.

[123] Barratt M, Huba M (1994). Factors related to international undergraduate student adjustment in an American community. Coll Student J.

[124] Cross SE (2016). Self-construals, coping, and stress in cross-cultural adaptation. J Cross-Cult Psychol.

[125] Masgoret A, Ward C, Sam DL, Berry JW (2006). Culture learning approach to acculturation. Cambridge Handbook of Acculturation Psychology.

[126] Lounsbury JW, Saudargas RA, Gibson LW (2004). An investigation of personality traits in relation to intention to withdraw from college. J Coll Student Devel.

[127] Mahmood H, Burke GB, Bista K (2014). An analysis of acculturative stress, sociocultural adaptation, and satisfaction among international students at a non-metropolitan university. Global Perspectives on International Student Experiences in Higher Education: Tensions and Issues.

[128] Ward C, Kennedy A (1994). Acculturation strategies, psychological adjustment, and sociocultural competence during cross-cultural transitions. Int J Intercult Relati.

[129] Wang CD, Mallinckrodt B (2006). Acculturation, attachment, and psychosocial adjustment of Chinese/Taiwanese international students. J Counsel Psychol.

[130] Ying Y (2005). Variation in acculturative stressors over time: a study of Taiwanese students in the United States. Int J Intercult Relat.

[131] Eustace RW (1999). Factors influencing acculturative stress among international students in the United States: Kansas State University.

[132] Zimet GD, Dahlem NW, Zimet SG, Farley GK (1988). The multidimensional scale of perceived social support. J Personality Assess.

[133] Kim B, Jee S, Lee J, An S, Lee SM (2017). Relationships between social support and student burnout: a meta-analytic approach. Stress Health.

[134] Canty-Mitchell J, Zimet G (2000). Psychometric properties of the Multidimensional Scale of Perceived Social Support in urban adolescents. Am jf Commun Psychol.

[135] Zimet G, Powell S, Farley G, Werkman S, Berkoff K (1990). Psychometric characteristics of the multidimensional scale of perceived social support. J Personality Assess.

[136] Chou K (2000). Assessing Chinese adolescents’ social support: the multidimensional scale of perceived social support. Personality Individ Differ.

[137] Dahlem NW, Zimet GD, Walker RR (1991). The multidimensional scale of perceived social support: a confirmation study. J Clin. Psychol.

[138] Creswell J (2001). Educational Research: Planning, Conducting, and Evaluating Quantitative and Qualitative Research, 4th Edition.

[139] Bowling A (2009). Research Methods In Health: Investigating Health and Health Services, 3rd Edition.

[140] Zamanzadeh V, Ghahramanian A, Rassouli M, Abbaszadeh A, Alavi-Majd H, Nikanfar A (2015). Design and implementation content validity study: development of an instrument for measuring patient-centered communication. J Caring Sci.

[141] Fiero M, Huang S, Oren E, Bell M (2016). Statistical analysis and handling of missing data in cluster randomized trials: a systematic review. Trials.

[142] Díaz-Ordaz K, Kenward M, Cohen A, Coleman C, Eldridge S (2014). Are missing data adequately handled in cluster randomised trials? A systematic review and guidelines. Clin Trials.

[143] Huh D, Flaherty BP, Simoni JM (2011). Optimizing the analysis of adherence interventions using logistic generalized estimating equations. AIDS Behav.

[144] Taylor A (2015). Standardised effect size in a mixed/multilevel model.

[145] Ferguson CJ (2009). An effect size primer: a guide for clinicians and researchers. Prof Psychol Res Pract.

[146] Nakimuli-Mpungu E, Musisi S, Wamala K, Okello J, Ndyanabangi S, Mojtabai R, Nachega J, Harari O, Mills E (2017). The effect of group support psychotherapy delivered by trained lay health workers for depression treatment among people with HIV in Uganda: protocol of a pragmatic, cluster randomized trial. JMIR Res Protoc.

[147] Cohen J (1962). The statistical power of abnormal-social psychological research: a review. J Abnorm Soc Psychol.

[148] Sink C, Stroh H (2006). Practical significance:the use of effect sizes in school counseling research. Prof School Couns.

[149] Durlak JA (2009). How to select, calculate, and interpret effect sizes. J Ped Psychol.

[150] Flemington T, Fraser J (2017). Building workforce capacity to detect and respond to child abuse and neglect cases: a training intervention for staff working in emergency settings in Vietnam. Int Emerg Nurs.

[151] Norberg M, Newins A, Jiang Y, Xu J, Forcadell E, Alberich C, Deacon B (2018). The scarier the better: maximizing exposure therapy outcomes for spider fear. Behav Cogn Psychother.

